# The effect of combined Action Observation Therapy with eccentric exercises in the treatment of mid-portion Achilles-tendinopathy: a feasibility pilot randomised controlled trial

**DOI:** 10.1186/s13102-022-00594-z

**Published:** 2022-11-29

**Authors:** Deirdre Ryan, Gráinne O’Donoghue, Ebonie Rio, Ricardo Segurado, Cliona O’Sullivan

**Affiliations:** 1 UCD School of Public Health, Physiotherapy and Sports Science, UCD, Dublin, Ireland; 2 School of Allied Health, La Trobe University Melbourne, Melbourne, Australia

**Keywords:** Mid-portion Achilles tendinopathy, Action Observation Therapy, Mirror neurone system, Neuroplasticity, Eccentric exercises, Motor control, Pain, Rehabilitation, Chronic musculoskeletal conditions

## Abstract

**Background:**

Mid-portion Achilles Tendinopathy (AT) is a common musculoskeletal condition with varying rehabilitation success rates. Despite the prevalence of this condition, a considerable proportion of individuals experience persisting pain and functional deficits. Current treatment approaches bias the biomedical model which emphasises physically treating and loading the tendon. Overall, there is a lack of consideration for the central nervous system that is commonly implicated in chronic injuries. The aim of this pilot study was to explore the feasibility of combining Action Observation Therapy (AOT), a treatment technique which targets central changes and influences motor learning, with eccentric exercises in the treatment of mid-portion AT. AOT involves the observation of movements and is commonly followed by the physical performance of these same movements.

**Methodology:**

This was a double-blinded randomised controlled pilot feasibility study. All participants underwent the 12-week Alfredson eccentric training protocol. The intervention group watched videos of the exercises prior to performing these exercises, whilst the control group watched nature videos before performing the same exercises. Study feasibility was the primary outcome measure, with the Victorian Institute of Sports Assessment- Achilles (VISA-A) selected as the primary clinical outcome measure.

**Results:**

Thirty participants were recruited, reflecting a 75% eligibility rate and 100% of eligible participants enrolled in the study. The retention rate at week 12 was 80%. At week six the mean VISA-A score improved by 18.1 (95% CI 10.2–26.0) in the intervention group and 7.7 (95% CI 0.3–14.9) in the control group, and 75% and 33% of participants in the intervention and control group respectively exceeded the minimal clinically important difference (MCID). At week 12 the mean VISA-A score from baseline improved by 22.25 (95% CI 12.52–31.98) in the intervention group and 16.5-(95% CI 8.47–24.53) in the control group, equating to 75% and 58% in each group respectively exceeding the MCID.

**Conclusion:**

The positive feasibility outcomes and exploratory data from the clinical outcome measures suggest that a larger scaled RCT is warranted to further investigate the impact of AOT in the rehabilitation of mid-portion AT.

*Trial registration* ISRCTN58161116, first registered on the 23/12/2020.

**Supplementary Information:**

The online version contains supplementary material available at 10.1186/s13102-022-00594-z.

## Introduction

The Achilles tendon is recognised as the strongest tendon within the human body [1]. Despite this, Achilles tendinopathy (AT) is common; 2.35 per 1000 years in adult populations [2] and up to 50% of runners can develop AT [3]. Clinically this condition presents as pain, morning stiffness and functional limitations, thus impacting the individuals physical abilities and quality of life. The result is impairments in body structure and function and limitations in activities and participant domains as classified by the International Classification of Functioning, Disability and Health (ICF) [4]. Risk factors associated with developing mid-portion AT include ageing, overuse, biomechanics, genetics, medication use, abnormalities on imaging such as the presence of vascularisation and increased tendon thickness and specific health conditions such as diabetes mellitus [5]. Structural characteristics span tendon thickening, collagen disorganisation, fibre separation, zones of neovascularisation and an increase in proteoglycans in the extracellular matrix [6], though these changes can exist without symptoms [7].

Mid-portion AT is recognised as a difficult condition to successfully rehabilitate and recovery can be slow or cease completely. Unpredictable clinical outcomes associated with this condition mean affected persons are at risk of long-term morbidity [8–10]. Exercise has the ability to address muscle changes [11–13] and is considered the optimal intervention for this condition [14]. Common rehabilitation practices for AT include graded loading protocols that potentially improve the mechanical properties and load tolerance of the tendon and kinetic chain [15, 16]. Loading can be concentric [17], heavy resistance training [18] or eccentric training which is the form of training currently most researched [19–22].

The brain and spinal cord are not structurally static and when musculoskeletal injury occurs, changes can arise in the structure, function and organisation of the nervous system [23]. Even in healthy adults, cortical representations of the body are continuously adapting in response to activity, behaviour and skill acquisition [23]. Until recent times it was considered that adaptions within a tendinopathic state were confined to the periphery, however it is now understood that both the peripheral and central systems are altered in tendinopathy [7], including lateral epicondyle, rotator cuff and patellar tendinopathy [24]. Corticospinal control directly influences tendon loading and motor performance [25], and alterations in corticospinal activity can negatively influence motor control as the primary somatosensory cortex processes sensory input and motor outputs are built upon this input. The body of literature detailing the effects of an altered somatosensory on motor output is ever expanding [26].

Reduced tendon stiffness in mid-portion AT demonstrating altered neuromuscular control and muscle activation patterns within the affected leg support the presence of central changes in persons with AT [27]. Additionally, gait parameters including double-limb support, step length and width, stride length and time and walking speeds have been shown to be reduced in runners with AT [28]. This same study further reported alterations in hip, knee and ankle moments in this population. A recent study concluded participants with chronic AT demonstrated motor dysfunction with single leg heel raises [29]. Soleus electromyographical activity has also been shown to be increased in individuals with AT as was flexor hallicus longus activity, a potential compensatory activation pattern [30]. Furthermore tactile acuity as measured with 2-point discrimination is correlated with somatosensory cortex reorganisation, has been shown to be significantly reduced over the affected tendon in AT populations [31].

**Fig. 1:**
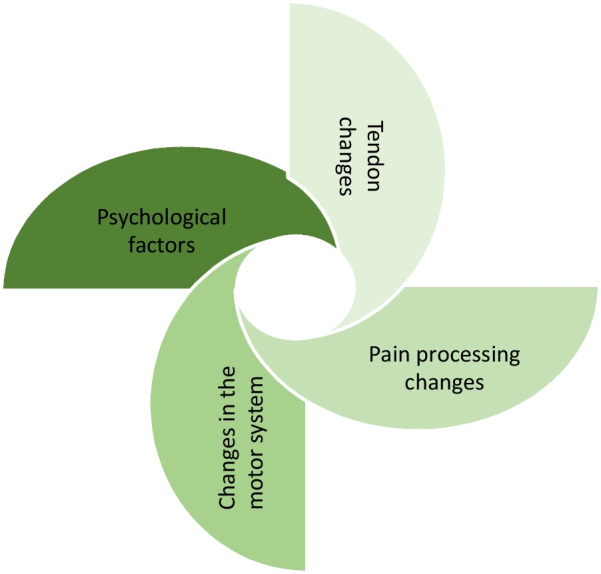
All potential systems invovled in AT

It is important to highlight that corticospinal changes in chronic musculoskeletal conditions are not confined to the motor system, and have correlations with pain too [32]. A study reporting decreased corticospinal excitability on the affected side in persons with rotator cuff tendinopathy, found chronicity of pain to be a relating factor to the lowered levels of excitability [33]. Signs of both peripheral and central sensitisation have been reported in chronic AT with both pain pressure thresholds and heat pain thresholds being lower on the affected and non-affected tendon in participants with AT as compared to controls [34]. Augmented central excitability has been suggested in the increased levels of pain catastrophizing and motor dysfunction that was reversed by local anaesthetic in persons with AT [29]. The above supports a multifactorial model for tendinopathy whereby local tendon pathology, an impaired motor system and changes in pain processing are involved [25, 35] (Fig. 1).

To improve outcomes for people with tendinopathy, all levels of dysfunction including changes that have occurred in the neural systems should be explored and addressed in rehabilitation programmes. One method that can facilitate neuroplasticity by influencing the primary cortex excitability is Action Observation Therapy (AOT) [36]. AOT involves the activation of the mirror neurons system (MNS), a network of neurones distributed throughout the brain. By observing movements, this specific MNS is activated promoting topographic cortical activity and plasticity within motor regions of the brain [37]. The core locations of this system like within the inferior frontal gyrus, the dorsal premotor cortex, the inferior parietal cortex, the supplementary motor area and the supplementary temporal gyrus [38].

A recent systematic review by members of this research group found strong to moderate evidence in support of AOT in the rehabilitation of individuals with stroke, Parkinson disease, multiple sclerosis and orthopaedic conditions [39]. The findings demonstrated improvements in motor and non-motor abilities including strength, walking, balance and disease specific health. An earlier systematic review found AOT to significantly improve body function and activity domains for upper and lower limb function in adults and children with stroke and cerebral palsy [40]. The more recent review also highlighted that AOT to be considerably unresearched in musculoskeletal conditions as compared to neurological conditions. The limited research available shows functional abilities and pain severity scores to improve post AOT in individuals with lower limb amputation [41], hip and knee arthroplasty [42–44] and chronic neck pain [45, 46]. Contrastingly, no additional benefits were found in the addition of AOT to rehabilitation programmes for persons with knee osteoarthritis [47].

The mismatch between current rehabilitation approaches for persons with mid-portion AT, and the evolving understanding of the role of the nervous system in tendinopathy needs to be addressed. The development of new rehabilitation pathways that combine a number of rehabilitation techniques is complex. In accordance with the Medical Research Council framework (MRC) for developing a complex intervention, feasibility and pilot studies are an integral step within this process [48]. Accordingly, the purpose of this study is to evaluate the feasibility of a future larger scale randomized controlled trial (RCT) in examining the effectiveness of AOT combined with an AT eccentric loading protocol in participants with mid-Portion AT.

The primary aim was to establish important feasibility outcome measures, pilot the acceptability of the study design and provide the necessary information to determine sample size calculations. The secondary aim was to explore the preliminary effects of intervention on the clinical outcome measures assessing central, physical and mental components.

## Methods

### Study design

This was a two-group parallel, double blinded randomised controlled feasibility pilot trial. The protocol for this study has been previously published and provides the study methods in detail [49]. The trial was first registered with the ISRCTN 58161116 on the 23/12/2020. The study findings are reported in accordance with the Consolidated Standards of Reporting Trials 2010 Statement [50] and the template for intervention description and replication guide [51].

### Study population

Full inclusion and exclusion criteria has been previously detailed [49]. A summary is provided in Table 1.

**Table 1:** Participant inclusion and exclusion criteria

Inclusion	Exclusion
• Male or female, aged 18–65 • Unilateral pain in mid-portion AT • Pain present for ≥ 3 months • Morning pain or stiffness • Access to a smart phone, tablet or laptop • Competent in English	• Suspicions of AT rupture • Previous AT surgery on affected side • Bilateral or insertional AT • Co-existing ankle or foot pathology or confounding lower limb injury • Systemic disease • Metabolic or endocrine disorders • Previous corticosteroid injection at/near site in last 3 months • Use of fluoroquinolone antibiotics in last 2 years • Participation in an active AT strengthening programme in the previous 3 months • Physical condition that prevented participation in an active exercise programme

### Recruitment and setting

The entire study was conducted online using zoom for all screenings and assessments. Participant recruitment ran from February to April 2021. Participants were recruited via social media (Twitter), posters emailed to Sports Clubs and posted around University College Dublin Campus. Study details were also circulated amongst staff. Potential participants contacted the lead researcher via phone or email and a time and date were promptly scheduled for the online screening assessment to be performed by a Physiotherapist (DR). Consent forms and further study information were emailed to all potential participants and signed consent forms were returned before screenings could commence.

### Randomisation and blinding

The randomisation and assignment of rehabilitation programmes were performed by two members of the research team with no involvement in the screening or assessment process. A randomisation list was generated using a web-based randomisation tool (randomizer.org). One member of the research team (GO’D) was responsible for study group allocation and relayed this information to the second member (CO’S) who created a Salaso account (an online application used by healthcare providers) for each participant and assigned the correct programme. All participants and the investigating researcher who administered the eligibility screening and all assessments were blinded to the group allocation.

### Advice and education

All participants were requested not to participate in other forms of AT rehabilitation or treatment for the duration of the study. At the end of the baseline screening session, all eligible participants underwent a tutorial with the investigating researcher regarding the exercise techniques for the Alfredson exercise protocol, photographs were shown and written instructions read aloud. Details regarding the Alfredson exercises are available in the protocol [49]. Participants were informed that a degree of pain or discomfort is normal, and to aim for the prescribed dosage pain permitting with acceptable pain levels being 4–5/10 on a scale where 10/10 is maximum levels of pain. Once the participants could do the exercises pain-free, they were encouraged to progress by introducing a weight. Five kg increments were recommended, however any increase in weight was allowed. A training calendar was sent to each participant to record all weight progressions. A support link for any technical difficulties with Salaso was also provided. Participants were informed to contact the lead researcher should any issues outside of technical issues arise and not to disclose the videos they are watching during this contact.

### Study interventions

All participants received an email link to a rehabilitation programme in Salaso, which could be downloaded onto a phone/tablet or accessed online via the webpage. Participants in the intervention group received two videos showing a healthy individual performing the 15 repetitions of each exercise. Participants in the control group received two dynamic landscape videos and a pdf containing photographs of an individual demonstrating both exercises accompanied by instructions on how to do the exercises. The individual in the videos and photos matched the participant for sex and side of injury and were captured from both a lateral and posterior view. The length of the videos were the same for both the control and intervention groups, and participants were instructed to watch video one before each set of exercise one and video two before each set of exercise two. All participants received educational emails at week 4 and week 8 in the study encouraging compliance and detailing the changes that were occurring in the tendon in response to the exercises.

### Outcome measures

#### Primary outcome measures

##### Rate of eligibility, enrolment, recruitment and retention

The rate of eligibility was deduced by the proportion of people who met the eligibility criteria versus the number who were screened for eligibility. The enrolment rate refers to the number of eligible participants who enrolled in the study. The recruitment rate was determined by the number of participants recruited divided by the number of months recruiting. The retention rate was the proportion of participants recruited who completed the 12 weeks of rehabilitation and the final assessment.

##### Adherence

Adherence was logged daily through Salaso and an overall percentage was calculated for the 12 weeks. Salaso does not accept logging retrospectively past midnight and so the training calendar was used as a secondary log for any completed sessions not logged in the application. Participants were also asked to give a verbal estimate of their exercise and video completion, where 100% was considered completing 3 sets of 15 repetitions of both exercises twice a day everyday and 100% for the videos was watching each video three times twice a day over the 12 weeks.

##### Acceptability of intervention

Acceptability was determined by the Patient Satisfaction Questionnaire that rated satisfaction levels with treatment as poor, moderate, good or excellent and the Patient Global Impression of Change scale (PGIC) which was scored on a 7-point likert scale ranging from very much improved to very much worse. Both measures were assessed at week 6 and week 12. Additionally, all participants were invited to participate in a semi-structured qualitative interview at the end of the study, exploring their experiences of the study. The findings of the qualitative exploration will be presented in a separate paper.

#### Secondary outcome measures

##### Clinical outcome measures

Twelve clinical outcome measures were selected and explanations and psychometric properties for these outcome measures are provide in the protocol [49]. Ten of the outcome measures address the core domains as outlined by the International Scientific Tendinopathy Consensus (ICON) group [52]; disability was assessed using the Victorian Institution Symptom Assessment- Achilles Questionnaire (VISA-A) which was the primary clinical outcome measure, pain over a specified time was assessed by the Numerical Pain Rating Score (NPRS) to score worst pain over the past week, pain on loading was assessed by the hop test, physical function capacity was assessed using the heel-raise test for endurance, participation was evaluated using the lower extremity functional scale (LEFS), participant rating of overall condition was assessed with the PGIC. Psychological factors were assessed with the Pain Catastrophising Scale, Tampa Scale for Kinesiphobia and pain self-efficacy questionnaire (PSEQ). Quality of life was assessed with the EQ-5D-5L. The additional outcome measures selected was the Widespread Pain Index (WPI) and symptoms severity to assess central pain processing and the Patient Satisfaction Questionnaire. The timelines of administration of the outcomes measures and ICF classifications are provided (Table 2). The International Physical Activity Questionnaire (IPAQ) was used to capture activity level profiles of the participants at the three time-points.

**Table 2:**
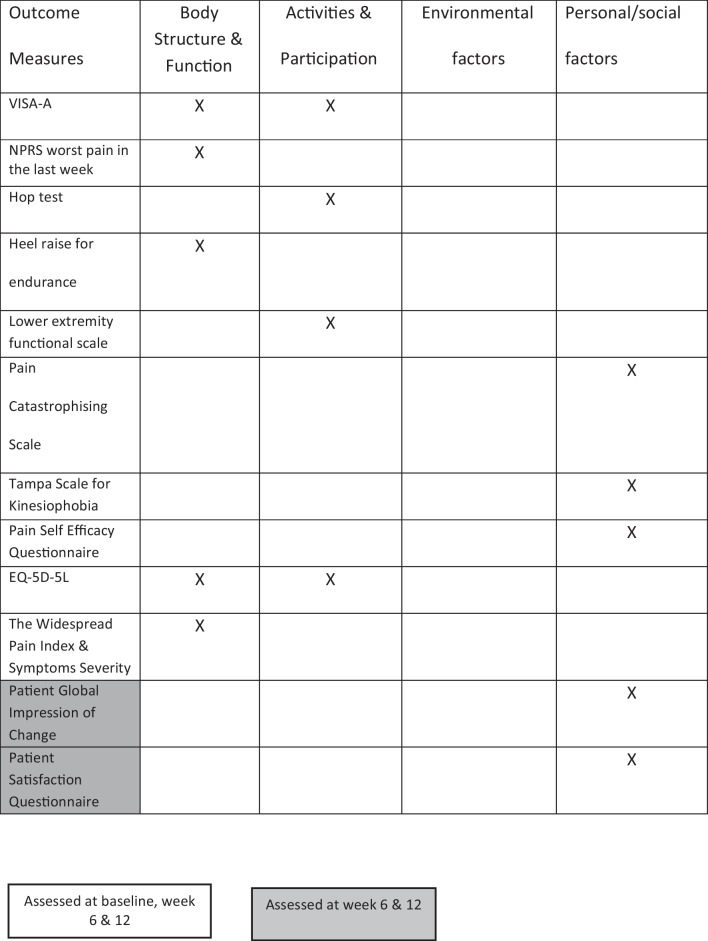
Classifying the clinical outcome measures under the ICF domains

##### Data analysis

Statistical analysis was performed using SPSS version 24. Feasibility outcomes were reported descriptively as percentages. Descriptive analysis of the quantitative data included mean scores and standard deviations and mean changes from baseline to 6 and 12 weeks with 95% confidence intervals (CI). Where minimal clinically important difference (MCID) were available for clinical outcome measures, the mean differences between baseline and week 6 and week 12 in each group were compared to these values. For exploratory purposes, the primary clinical outcome measure (VISA A) was analysed using an analysis of covariance (ANCOVA) with baseline scores the covariate, group allocation the fixed factor and the follow-up assessment the dependent variable. A sample size calculation was performed using the web-based calculator designed by Vanderbilt University (https://vbiostatps.app.vumc.org/), selecting the VISA-A MCID and pooled standard deviation from both groups at week 12.

## Results

### Participants

**Fig. 2:**
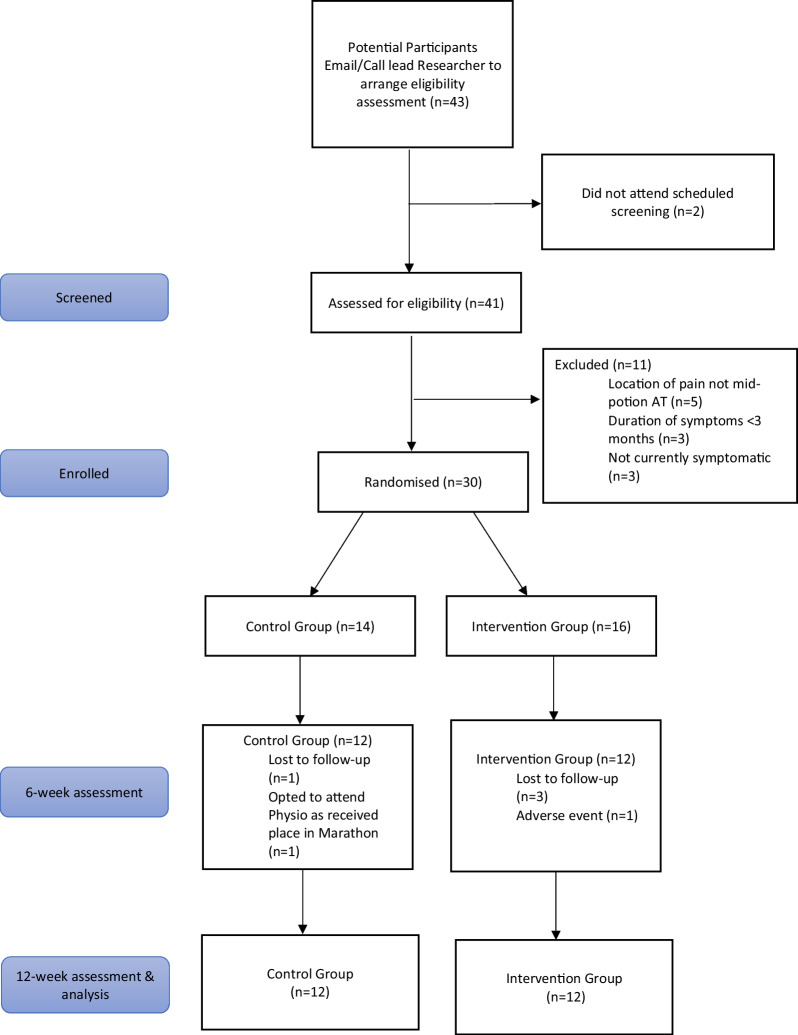
Prisma flow diagram of the screening process

Between February and April 2021, 43 participants contacted the lead researcher to arrange a screening session. A total of 30 participants were enrolled in the study, exceeding the 12 participants minimum per group outlined in the protocol [49], 14 participants in the control group and 16 participants in the intervention group (Fig. 2). Upon visual inspection there were clinically no differences in baseline characteristics between the groups, the control group however did have a longer duration of symptoms [53] (Table 3) (Additional file 1). Physical activity profiles as defined by the IPAQ revealed a greater proportion of participants in the control group had high physical activity levels and a greater proportion of participants in the intervention group had moderate physical activity levels. Two of the participants lost after baseline in the intervention group were in the high physical activity category (Tables 3 and 4).

**Table 3:** Participant Characteristics at baseline [53]

	Control Group (14)	Intervention Group (16)
Age (Years), Mean (SD)	45 (9.4)	41.5 (8.2)
Female/Male	7/7	8/8
Height (cm), Mean (SD)	173 (8.7)	176 (9.5)
Weight (kg), Mean (SD)	76 (10.7)	80 (15.8)
BMI, MEAN (SD)	25.4 (3.3)	25.9 (5.1)
Side of injury (L/R)	7/7	10/6
Duration of Symptoms (months)	14 (16.2)	10 (7.2)
Flouroquinolone antibiotics (Y/N)	0/14	0/16
Corticosteroids (Y/N)	0/14	2/14
Anti-inflammatory medication (Y/N)	4/10	6/10
Statin medication (Y/N)	0/14	0/16
Diabetes medication (Y/N)	0/14	0/16
Allopurinol medication (Y/N)	0/14	0/16
Aromatrose inhibitors	0/14	0/16
Physical Activity Levels (active/inactive)	14/0	12/4
International Physical Activity Questionnaire (High/Moderate/Low)	7/7/0	6/9/1
Presence of comorbidities (Y/N)	1/13	1/15
Inflammatory or rheumatoid arthritis (Y/N)	0/14	0/16
Psoriasis (Y/N)	1/13	1/15
Inflammatory bowel or eye disease (Y/N)	0/14	0/16
Ankylosing spondylitis (Y/N)	0/14	0/16
Gout (Y/N)	0/14	0/16
Diabetes 1 or 2/ impaired glucose sensitivity (N)	0/14	0/16
Hypertension or cardiac disease (Y/N)	1/13	1/15
Hypertriglyceridemia/ high cholesterol (Y/N)	2/12	0/16
Familial hypercholesterolemia (Y/N)	5/9	8/8
Collagen disorders (Y/N)	0/14	0/16
Fibromyalgia (Y/N)	0/14	0/16
Imaging (N/MRI/US)	11/2/1	16/0/0
Prior History of Tendinopathy (N/Achilles/Patellar)	9/4/1	10/5/1

**Table 4:** Clinical outcome measures & the IPAQ

Outcome	Control group		Intervention group		
		% exceeding MCID		% exceeding MCID	ANCOVA
VISA-A					
Baseline	68.2 (17.2)		64.2 (13.6)		
Week 6	75.8 (13.0)		82.3 (10.3)		
Week 12	84.7 (11.5)		86.4 (18.2)		
Mean difference at 6 weeks (95% CI)	7.6 (0.28 to 14.9)	33	18.1 (10.2 to 26.0)	75	*p* = 0.037 η^2^ = 0.192
Mean difference at 12 weeks (95% CI)	16.5 (8.5 to 24.5)	58	22.3 (12.5 to 32.0)	75	*p* = 0.447 η^2^ = 0.028
Left heel-raises (n)					
Baseline	20.8 (8.7)		25.9 (11.9)		
Week 6	25.0 (8.8)		30.2 (15.5)		
Week 12	28.8 (11.6)		38.2 (18.1)		
Mean difference at 6 weeks (95% CI)	4.2 (0.6 to 7.7)		4.3 (− 1.0 to 9.5)		
Mean difference at 12 weeks (95% CI)	7.9 (2.9 to 12.9)		12.2 (3.4 to 21)		
Right heel-raises (n)					
Baseline	22.6 (8.2)		25.3 (10.6)		
Week 6	24.8 (9.5)		28.7 (10.9)		
Week 12	28.7 (10.6)		33.9 (14.6)		
Mean difference at 6 weeks (95% CI)	2.2 (− 1.6 to 6.0)		3.42 (− 0.9 to 7.7)		
Mean difference at 12 weeks (95% CI)	6.1 (0.5 to 11.6)		8.6 (3 to 14.4)		
Left Hop Test (NPRS)					
Baseline	4.8 (2)		4.1 (1.9)		
Week 6	2.29 (2.43)		2.7 (2.2)		
Week 12	1.14 (1.86)		0.8 (1.6)		
Mean difference at 6 weeks (95% CI)	− 2.6 (− 4.5 to − 0.7)		− 1.1 (− 3.4 to 1.2)		
Mean difference at 12 weeks (95% CI)	− 3.4 (− 5.9 to − 1.0)		− 3.3 (− 5.9 to − 0.8)		
Right hop test (NPRS)					
Baseline	3.3 (1.5)		5.1 (1.7)		
Week 6	1.6 (2.3)		1.7 (3.6)		
Week 12	0.5 (1.0)		1.7 (2.9)		
Mean difference at 6 weeks (95% CI)	− 0.8 (− 1.8 to .24)		− 3.3 (− 6.5 to − 0.2)		
Mean difference at 12 weeks (95% CI)	− 2.3 (− 5.5 – 1.0)		− 3.8 (− 6.7 to − 1.0)		
NPRS					
Baseline	5.6 (1.9)		5.3 (2.3)		
Week 6	4.4 (2.1)		3.3 (2.4)		
Week 12	1.8 (1.29)		1.3 (2.1)		
Mean difference at 6 weeks (95% CI)	− 1.2 (− 2.4 to 0.1)	58	− 1.9 (− 3.7 to − 0.2)	50	
Mean difference at 12 weeks (95% CI)	− 3.8 (− 5.2 to − 2.5)	75	− 4 (− 5.7 to − 2.3)	83	
LEFS					
Baseline	78.9 (15.2)		77.0 (16.6)		
Week 6	84.5 (12.2)		88.7 (9.6)		
Week 12	94.5 (7.41)		92.9 (8.5)		
Mean difference at 6 weeks (95% CI)	5.6 (1.5 to 9.8)	17	11.7 (4.1 to 19.3)	42	
Mean difference at 12 weeks (95% CI)	15.6 (8.7 to 22.6)	66	16 (8.3 to 23.6)	58	
PSEQ					
Baseline	52.8 (7.4)		52.75 (7.0)		
Week 6	55.6 (6.1)		54.7 (4.7)		
Week 12	57.3 (4.3)		56.7 (4.6)		
Mean difference at 6 weeks (95% CI)	2.8 (0.1 to 5.6)	0	2.8 (− 1.1 to 6.6)	25	
Mean difference at 12 weeks (95% CI)	4.6 (1.3 to 7.9)	17	4.8 (1.5 to 8.0)	8	
EUROQOL-5D VAS					
Baseline (95% CI)	83.9 (78.9 to 88.8)		80.0 (72.3 to 85.7)		
Week 6 (95% 5CI)	85.0 (80.1 to 89.9)		82.5 (73.9 to 91.1)		
WEEK 12 (95%CI)	87.0 (82.7 to 91.5)		82.4 (74.2 to 90.6)		
PGIC					
Very much improved week 6/week 12(N)	1/6		4/8		
Much improved week 6/week 12 (N)	6/5		4/1		
Minimally improved week 6 /week 12 (N)	5/1		2/2		
No change week 6/week 12 (N)	0/0		1/0		
Minimally worse week 6/week 12 (N)	0/0		1/1		
Satisfaction Q					
Excellent week 6/week 12 (N)	5/7		8/9		
Good week 6/week 12 (N)	4/3		1/1		
Moderate week 6/week 12 (N)	3/2		3/1		
POOR week 6/week 12 (N)	0/0		0/1		
IPAQ					
Week 6 high/moderate/low	6/6/0		3/8/1		
week 12 high/moderate/low	8/3/1		3/8/1		

		N exceeding threshold		N exceeding threshold
PCS				
Baseline	14.7 (14.0)	3	15.6 (8.3)	1
Week 6	8.4 (9.1)	1	6.6 (3.8)	0
Week 12	2.9 (2.2)	0	3.7 (2.8)	0
Mean difference at 6 weeks (95% CI)	− 6.3 (− 12.1 to − 0.4)		− 9.0 (− 14.4 to − 3.7)	
Mean difference at 12 weeks (95% CI)	− 11.8 (− 20.9 to − 2.6)		− 11.9 (− 17.8 to − 6.0)	
TSK				
Baseline	34.6 (7.6)		38.4 (6.1)	
Week 6	33.3 (4.5)	2	34.0 (6.3)	4
Week 12	28.5 (5.2)	1	30.8 (6.2)	3
Mean difference at 6 weeks (95% CI)	− 1.3 (− 4.4 to 1.9)		− 4.4 (− 6.2 to − 2.7)	
Mean difference at 12 weeks (95% CI)	− 6.1 (− 9.9 to − 2.3)		− 7.67 (− 11.3 to − 4.0)	

Results are presented as mean (std dev), mean difference (95% CI) and proportion exceeding MCID, η^2^ = partial eta squared is provided for the primary clinical outcome measure.

*N* number,* LEFS* Lower Extremity Functional Scale,* PSEQ* Participant Self-Efficacy Questionnaire,* PGIC* Participant Global Impression of Change,* Q* Questionniare,* IPAQ* International Physical Activity Questionnaire,* PCS* Pain Catastrophising Scale,* TSK* Tampa Scale Kinesiophobia

### Rate of eligibility, enrolment, recruitment and retention

A total of 73% of the participants screened were eligible for enrolment. The enrolment rate was 100%. The average recruitment rate was 10 participants per month, ranging from 8 to 13 participants. The retention rate was 80%, with 20% of participants being lost between baseline and week 6 (Fig. 2). One participant was lost to an adverse event which prevented physically performing the eccentric exercises (discomfort in the plantar fascia on the symptomatic side). It was not established whether the onset of injury was related to the exercises.

### Exercise adherence

Participant reported exercise adherence over the 12 weeks was 72% (95%CI 55.9–88.2) in the intervention group and 79.5% (95%CI 66.8–92.1) in the control group. Participant reported video adherence over the 12 weeks was 50% (95%CI 34.8–65.2) in the intervention group and 67.7% (95% CI 52–83.4) in the control group. The actual logged adherence in Salaso was 50.3% (95% CI 31.8–68.7) in the intervention group and 31.3% (95% CI 16.8–45.8) in the control group.

### Acceptability

At week 6 satisfaction with treatment levels were either excellent or good in 75% of participants in both groups, with 66.7% of participants in the intervention group and 41.7% of participants in the control group rating satisfaction levels as excellent. At week 12 satisfaction levels were either excellent or good in 83% of participants in both groups, 75% in the intervention group and 58.3% in the control group rated their satisfaction levels as excellent. In the PGIC at week 6, 33.3% of participants in the intervention group and 8.3% of participants in the control group classified their change in symptoms as very much improved. This increased to 67% of participants in the intervention group and 50% in the control group at week 12.

### Sample size calculation

The pooled standard deviation in the VISA-A at week 12 was 14 and an MCID of 10 was selected. A future RCT would need 32 participants per treatment arm to achieve 80% power using type I set at 0.05.

### Clinical outcome measures

#### Primary clinical outcome measure

**Fig. 3:**
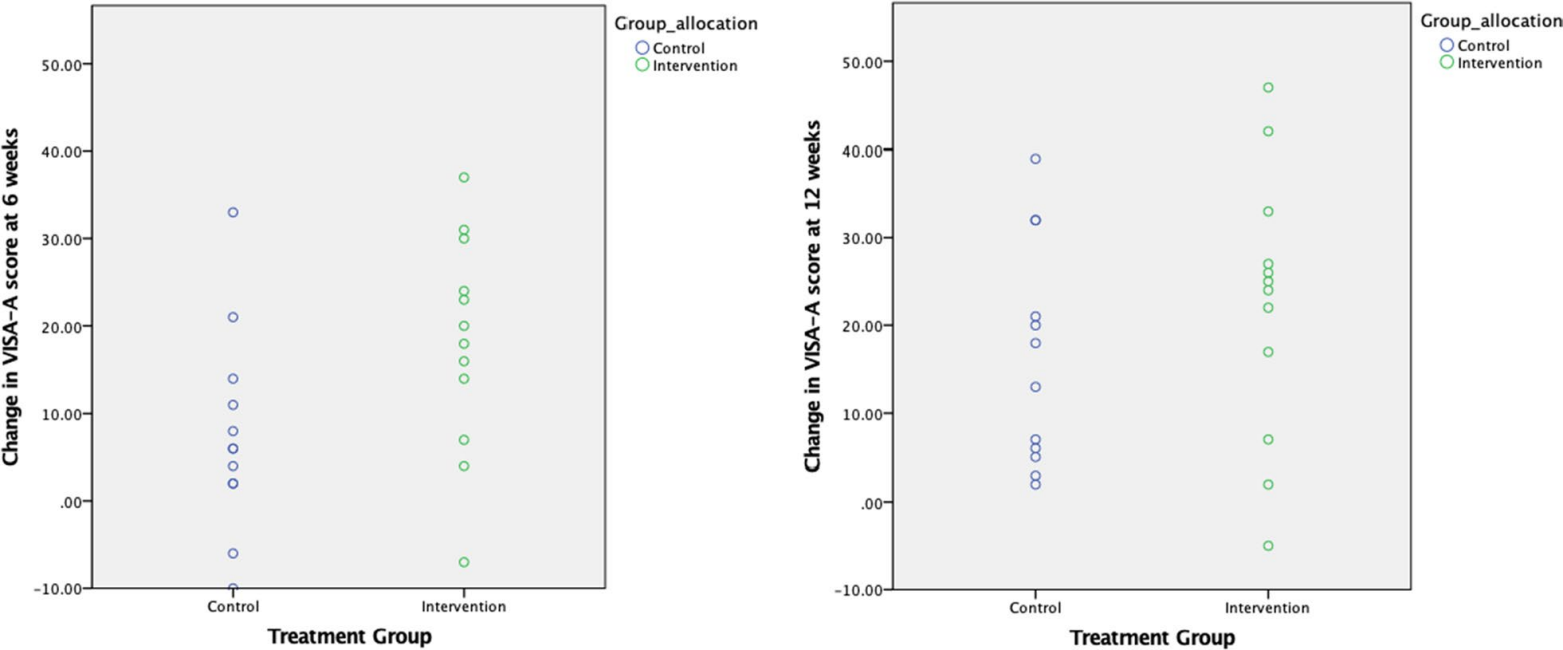
Individual participant changes in VISA-A scores at 6 & 12 weeks

At week 6 the mean VISA-A score improved by 18.1 ( 95% CI 10.2 to 26.0) in the intervention group and 7.6 (95%CI 0.3 to 14.9) in the control group (Table 4) (Fig. 3). At week 6, 75% participants in the intervention group exceeded the MCID of 10 points compared to 33% of participants in the control group. At week 12 the mean VISA-A score from baseline improved by 22.3 (95% CI 12.5 to 32.0) in the intervention group and 16.5 (95% CI 8.5 to 24.5) in the control group, equating to 75% in the intervention group and 58% in the control group exceeding the MCID (Table 4) (Fig. 3).

#### Secondary clinical outcome measures

Improvements in scores were seen in both groups, however a greater improvement in mean difference (95% CI) in the intervention group was seen at both week 6 and week 12 in the worst NRPS over the past 7 days, NPRS on the right hop test, the LEFS, the Pain Catastrophising Scale and the Tampa Scale for Kinesiophobia (Table 4). The margin of the group differences in scores were greater at week 6 for these outcome measures (Table 4). The improvements in the heel-raises were greater in the intervention group at week 6 and week 12, and the margin of this difference continued to increase at week 12 (Table 4). The mean difference for NPRS on the left hop test was greater in the control group at week 6 and week 12, with the between-group differences minimal at week 12. The score improvements in mean differences in the PSEQ were greater at week 6 in the control group and greater in the intervention group at week 12, with the margin of differences being small at both time-points (Table 4).

**Fig. 4:**
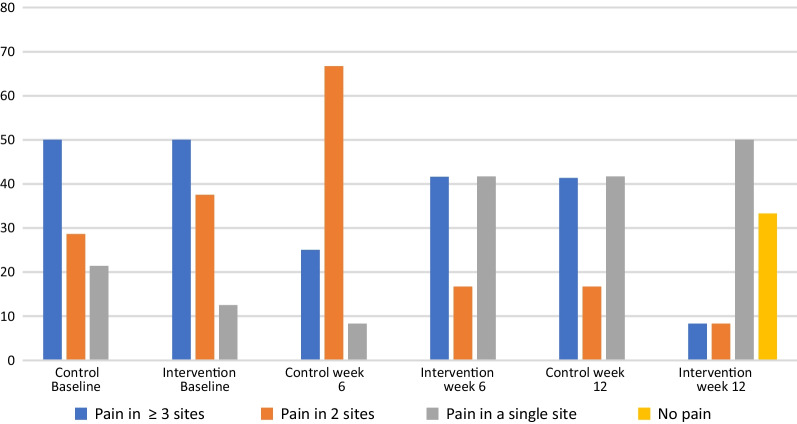
Widespread pain index detailing the number of bodily pain sites

In terms of patient profiling at baseline, three participants in the control group and a single participant in the intervention group exceeded 30 points in the Pain Catastrophising Scale, thus indicating the presence of catastrophising thoughts amongst these participants. At week 6, a single participant in the control group alone scored over 30 points. At week 12 no participant in either group demonstrated catastrophising thoughts. The number of people scoring 37 or higher and therefore displaying kinesiophobia decreased by 31.3% in the intervention group and 41.7% in the control group over the 12 weeks. Three participants demonstrated central sensitisation as classified by the WPI and symptom severity scores; one participant was in the intervention group and was lost to follow-up after the baseline assessment, a second participant in the control group scored above the threshold at all three time-points whilst the final participant in the intervention group only scored above the threshold at the final assessment, it important to note that this participant did not actively participate in the trial after the week 6 mark. The proportion of participants experiencing pain in a single site, two sites or three or more sites as per the WPI is illustrated (Fig. 4).

**Fig. 5:**
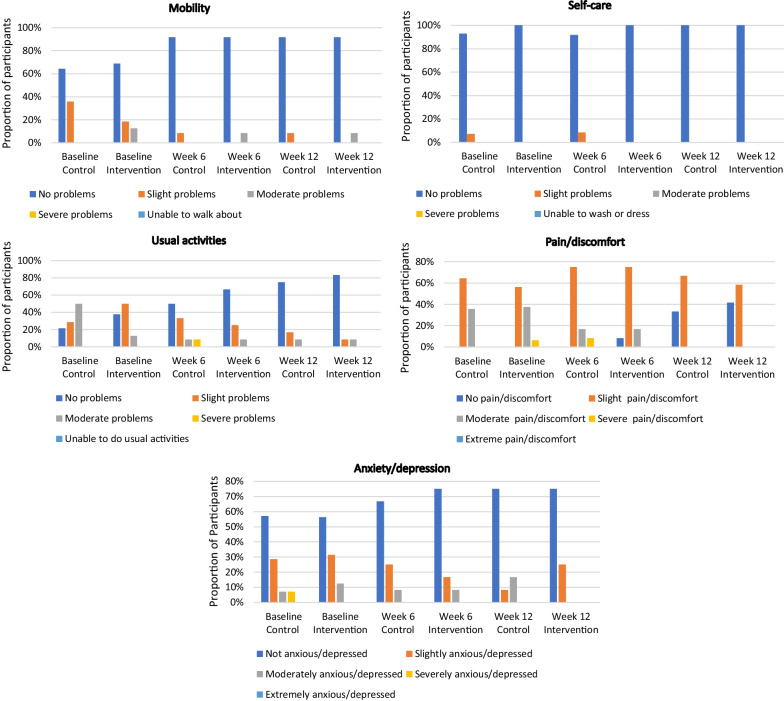
Illustrating proportion of participant scores in the five domains of the EQ-5D-5L

At baseline, the dimensions of health most affected was pain/discomfort followed by usual activities, anxiety/depression and mobility as assessed in the EQ-5D-5L. Self-care appears to be the most minimally impacted domain (Fig. 5). At week 6 improved health is seen in 75% of participants in the intervention group and 58.3% of participants in the control group whereby at least one dimension has improved and no dimension has dis-improved. At week 12 overall health was improved from baseline in 100% of participants in the intervention group and 71.4% of participants in the control group.

## Discussion

This is the first study to pilot and establish the feasibility in combining AOT with eccentric exercises in the rehabilitation of mid-portion AT. The findings demonstrate high recruitment potential with the total number of participants recruited in three months. The progression criteria as previously outlined in the protocol [49] was met, with the retention rate reaching the 80% threshold and the 100% enrolment rate surpassing the minimum enrolment rate previously outlined, therefore indicating the feasibility of a full-scale RCT. The participant baseline characteristics in terms of age, weight and height are similar to that reported in other pilot studies investigating mid-portion AT but the duration of symptoms appears to be shorter [54–57].

Whilst this is a pilot feasibility study, therapeutic measures were explored and establishing the adherence with such measures was important in order to explore any preliminary effects of treatment. Adherence with health interventions is complex, particularly for individuals with chronic conditions, many inter-dependent factors are at play including characteristics of the patient, the treatment, the condition, the relationship between the healthcare provider and patient along with the clinical setting [58]. Exercise adherence levels were in the 70 percentile for both groups. Subjectively reported video adherence was nearly 20% higher in the control group, interestingly the objective figure recorded in Salaso was substantially less. For the intervention group, the reported and recorded figure were both 50%. The discrepancy between the recorded and reported figures in the control group may be due to a subjective over-estimation of exercise adherence or perhaps incorrect use of the log function in Salaso. A previous systematic review found the provision of Additional file 1 such as audio or videos had a positive effect on increasing exercise adherence in patients with chronic musculoskeletal pain, this could explain the increased logged adherence in the intervention group [59]. No minimum threshold of adherence has currently been established for AOT. This is the first study exploring AOT and tendinopathy, therefore how much AOT is needed or whether a minimum threshold exists was unknown, hence the prescription of AOT prior to every set may be over-prescribing. A larger RCT would allow different dose prescriptions of AOT to be compared, which would allow refinement of the intervention going forward, an important aspect of complex interventions [48].

Acceptability of intervention can dictate whether an intervention is successful or not. Many factors can influence perceived acceptability of interventions including the content, context and quality of care as well as expectations [60]. In terms of treatment acceptability, similar positive results are seen in both groups at week 6 and week 12, with the intervention group having a higher proportion of participants rating their satisfaction levels as excellent. As for the PGIC, both groups demonstrated positive percentages of participants who rated themselves as either very much improved or improved, again the proportion of very much improved alone was higher in the intervention group at both time-points. The results from the qualitative study will provide a more in-depth understanding into the acceptability of this study.

Looking at the clinical outcome measure mean changes at week 6, a total of 9.5 (hopping and heel-raises 0.5 for each side) of the outcome measures revealed superior results in the intervention group, including the VISA-A, the NPRS, the LEFS, the Pain Catastrophising Scale, the Tampa Scale for Kinesiophobia, heel-raises, pain on hopping on the right side, PGIC, the Patient Satisfaction Questionnaire and the EQ-5D-5L. Similar changes were seen in both groups at week 6 in the PSEQ, whilst superior results were seen in the control group in pain on hopping on the left side. The WPI was used to capture participants presenting with central sensitisation and mean changes were not compared in this capacity. The greater results seen in the VISA-A, the selected primary clinical outcome measure, in the intervention group equated to 75% of participants exceeding the MCID at week 6. The VISA-A measures disability, pain and ability to participate in everyday tasks and a pattern in the results is evident as worst pain scores in the past 7 days and functional ability as measured by the NPRS and the LEFS respectively were also better in the intervention group at week 6. The central nervous system has previously been suggested as the cause for positive adaptions in the short-term [61]. The cause for this positive change may be due corticospinal changes. This is unsurprising as we know that AOT results in modulation of corticospinal excitability [62, 63], thus facilitating neuroplasticity. It has previously been established that activation of the MNS can facilitate motor learning and improve pain [39, 64–66]. The pain and motor improvements as captured by the patient reported outcome measures along with the physical tests in the intervention group may be in part due to the activation of the MNS, explaining the superior results seen in this group. Preliminary impressions would be that the dysfunctional neuromuscular adaptions in response to pain and injury that can occur in mid-portion AT were mediated through AOT and its influence on corticospinal activity.

At week 12, whilst there is a preservation of the positive changes noted at week 6 in the intervention group, there appears to be a general slowing down in the rate of improvement in the intervention group and a continued improvement in the control group, with the between-group differences now being marginally in favour of the intervention group in the NPRS, the LEFS, the Pain Catastrophising Scale, the PGIC and the Patient Satisfaction Questionnaire. Greater score differences were seen in the VISA-A and Tampa Scale for Kinesiophobia favouring the intervention group, however these are to a lesser degree than those seen at week 6. The results in the EQ-5D-5L continue to be superior by a notable percentage in the intervention group. Similar results continue to be seen in both groups in the PSEQ, whilst differences in pain on hopping on the left-side has decreased between the groups as this continued to be the only measure where the control group scored better than the intervention group, whilst pain on hopping scores on the right-hand side continued to be better in the intervention group, although it is important to note that minimal further gains were seen between week 6 and week 12. The results in the heel-raises continued to be notably better in the intervention group. It is not surprising that the muscle endurance gains had continued in the latter half of the study as this is when the musculotendinous adaptations occur, whereas earlier improvements after eccentric training were likely due neuronal adaptations [67]. To conclude at this time-point, results are marginally better in the intervention group in 9.5 of the outcome measures and much better in two of the measures, with the between-group differences smaller at this timepoint. Understanding when best does AOT contribute to change is an important consideration going forward and is an important aspect within the MRC framework [48]. The decline in the rate of improvement between week 6 and week 12 should be considered going forward, perhaps AOT is better placed in the first half of a 12 week rehabilitation programme.

The outcome measures included in this study possess sound psychometric properties [48]. Wherever possible mean changes were compared to MCID values that helped establish a therapeutic threshold and clinical relevancy [68]. It is worth mentioning that in certain instances the baseline mean scores were so high that there was no room for improvements to meet the MCID values, for example in the PSEQ, mean baseline values were 52.8 (7.4) in control group and 52.75 (7.0) in the intervention group and a score change of 11 points is required to detect an MCID. The lack of detection of an MCID should be considered in light of mean values presented. These baseline PSEQ values were similar to those reported in other studies investigating AT [69].

In the selection of outcome measures, three scales were selected under the psychological domain; the Pain Catastrophising Scale, PSEQ and the Tampa Scale for Kinesiophobia. Science has highlighted the contribution of maladaptive psychological features towards ongoing chronic pain, motor dysfunction and delayed recovering from physiotherapy rehabilitation [29, 70, 71]. Yet to date, psychological factors are largely unexplored in persons with AT. A recent scoping review under-taken by two of the authors identifying outcome measures used in intervention studies in the rehabilitation of AT, noted not one of the 38 included studies assessed psychological factors [72]. Therefore, it was important for psychological factors to be recognised along with the other systems (Fig. 1). As the numbers included in this study were too small to truly explore the impact of AOT on eccentric exercises in subgroups of participants presenting with low levels of self-efficacy or high levels of kinesiophobia or catastrophisation, analysis of larger numbers in a full-scale RCT would be able to investigate this further. A recent study reported that participants with higher levels of kinesiophobia had higher levels of pain catastrophisation, higher levels of pain in their AT at rest and with activities, along with a decreased willingness to perform Achilles tendon loading activities [73]. As the reconceptualization of maladaptive fear avoidance beliefs is essential to reduce perceived threat of fear and pain, viewing an individual perform the rehabilitation movements safely could help with this process, altering the patients expectation from the movements they were to perform prior to the execution [74]. Participants were instructed that an element of pain was normal and expected when performing the exercises, and pain levels were to be used as a guide for progression. In this regard pain was encouraged and welcomed therefore re-shaping the participants beliefs and expectations surrounding the negativity commonly associated with pain.

The strengths of this study include the high levels of acceptability, good retention rates, excellent enrolment rates and the detailed description of included participants [53]. The selection of outcome measures are a further strength, as psychological factors were assessed and at least one outcome measure was selected from each of the core domains as outlined by the ICON group [52]. The MRC recognises that a crucial aspect of evaluation design is indeed in the selection of outcome measures [48]. Following the ICON international standards facilitates homogeneity of results allowing comparisons amongst trials going forward. The results of this study have informed the necessary sample size needed to conduct a full-scale study, providing valuable information. The online aspect meant participants could do their exercises at a time and place of convenience and did not have to travel which was cost-effective. Bias was minimised throughout the study with both the participants and assessor blinded to group allocations. However, the duration of symptoms was 14 months in the control group and 10 months in the intervention group, this difference could potentially have been a source of bias between the groups. Study limitations were the high volume of AOT prescribed likely negatively impacting adherence to the intervention and the fact that Salaso cannot record past midnight was an inconvenience for participants. A single adverse event was noted, although the participant did not specify that the plantar fascia tightness was a result of the exercises. Additionally, given the small sample size stratification of participants for potential confounding factors (such as weight or activity levels) was not able to be performed. Similarly, there were not adequate numbers to truly capture and explore the relationship of fear of movement, catastrophisation, central sensitisation and AOT.

In conclusion, this pilot feasibility study has demonstrated that a full-scale RCT is feasible to investigate the effect of AOT combined with eccentric loading exercises in the treatment of mid-portion AT. This would allow the intervention to be further explored for potential clinical use and assess factors such as dose response.

## Supplementary Information

**Additional file 1**. Participant baseline demographics as recommended by the ICON group [53].

## Data Availability

All data generated or analysed during this study are included in this published article (and Additional file 1).

## Abbreviations

AT: Achilles tendinopathy
AOT: Action Observation Therapy
CI: Confidence interval
ICF: International Classification of Functioning, Disability and Health
ICON: International Scientific Tendinopathy Consensus
LEFS: Lower extremity functional scale
MCID: Minimum clinically important difference
MNS: Mirror neurone system
MRC: Medical Research Council
NPRS: Numerical pain rating scale
PGIC: Patient global impression of change
PSEQ: Participant self-efficacy questionnaire
VISA-A: Victorian Institute of Sports Assessment-Achilles
WPI: Widespread pain index
