# Supplementary material for: The effect of combined Action Observation Therapy with eccentric exercises in the treatment of mid-portion Achilles-tendinopathy: a feasibility pilot randomised controlled trial
Source: BMC Sports Sci Med Rehabil. 2022 Nov 29;14:201. doi: 10.1186/s13102-022-00594-z (PMC9706872; doi:10.1186/s13102-022-00594-z)
Supplement: Supplementary file 1 — Additional file 1. Participant baseline demographics as recommended by the ICON group [53]. [file 13102_2022_594_MOESM1_ESM.docx]

| **Participant Demographics** | |
| --- | --- |
| Age |  |
| Sex |  |
| Body Mass |  |
| Height |  |

**Additional File 1;** Participant baseline demographics as recommended by the ICON group [53]

| **General Health** | |
| --- | --- |
| Medication: |  |
| Fluoroquinolone antibiotics (eg ciprofloxacin) |  |
| Corticosteroids |  |
| Anti-inflammatory medication |  |
| Statin medication (cholesterol) |  |
| Medication for diabetes |  |
| Allopurinol (gout medication) |  |
| Aromatase inhibitors (used post breast cancer) |  |
| Physical Activity Level |  |
| Presence of Co-morbidities: |  |
| Inflammatory or rheumatoid arthritis |  |
| Psoriasis |  |
| Inflammatory bowel or eye disease |  |
| Ankylosing Spondylitis |  |
| Gout |  |
| Diabetes (type 1 or 2) or impaired glucose sensitivity |  |
| Hypertension or cardiac disease |  |
| Hypertriglyceridemia and/or high cholesterol |  |
| Familial hypercholesterolemia |  |
| Collagen disorders such as Ehlers Danlos syndrome |  |
| Fibromyalgia |  |
| Prior History of Tendinopathy ? |  |

| **Tendinopathy Descriptors** | |
| --- | --- |
| Duration of Symptoms |  |
| Severity of symptoms |  |
| Level of disability |  |
| Location of symptoms |  |
| Loading tests used |  |

| **Recruitment and Screening** | |
| --- | --- |
| Recruitment source |  |
| Details of recruitment strategy |  |
| Use of imagine to screen |  |
